# In vivo characterization of abdominal aortic aneurysms using an elastin specific molecular MR probe

**DOI:** 10.1186/1532-429X-16-S1-O13

**Published:** 2014-01-16

**Authors:** Marcus R Makowski, Andrea J Wiethoff, Hans U Ebersberger, Christian H Jansen, Ulrike Blume, Alice Warley, David Onthank, Richard R Cesati, Reza Razavi, Mike Marber, Tobias Schaeffter, Simon Robinson, Rene Botnar

**Affiliations:** 1 Kings College London, London, UK; 2 Philips Healthcare, London, UK; 3 Lantheus Medical Imaging, North Billerica, Massachusetts, USA; 4 Department of Radiology, Charite, Berlin, Germany

## Background

Rupture of abdominal aortic aneurysms (AAAs) is the third most common cause of death in cardiovascular diseases. Despite this high significance, there is still controversy regarding the management of AAAs, as diameter is currently the only accepted parameter to assess risk of rupture. Elastin is the key protein for maintaining stability of the aortic wall. The aim of this study was to evaluate a novel small-molecular-weight elastin-specific MR probe for the in-vivo assessment of aortic wall integrity in AAAs.

## Methods

ApoE-knockout-mice (ApoE-/-) were infused with angiotensin-II (Ang-II) for up to four weeks (1000 ng/kg/min) to induce AAA formation. An elastin-specific MR probe (Lantheus Medical Imaging, USA) was administered 1, 2, 3 and 4 weeks following Ang-II infusion. Mice were scanned at each time point pre, post control agent (Gd-DTPA) and after administration of the elastin-specific probe. Imaging was performed using a 3T Philips-Achieva MR-scanner equipped with a microscopy coil. Imaging parameters of 3D IR-MRI: spatial resolution = 0.1 × 0.1 mm, 0.5 mm slice-thickness, TR/TE = 28/8.2 ms. Additionally 3D-T1 mapping was performed. Ex-vivo tissue samples were analysed by inductively-coupled-plasma mass-spectroscopy (ICP-MS), histological staining and electron microscopy.

## Results

**Figure 1:**
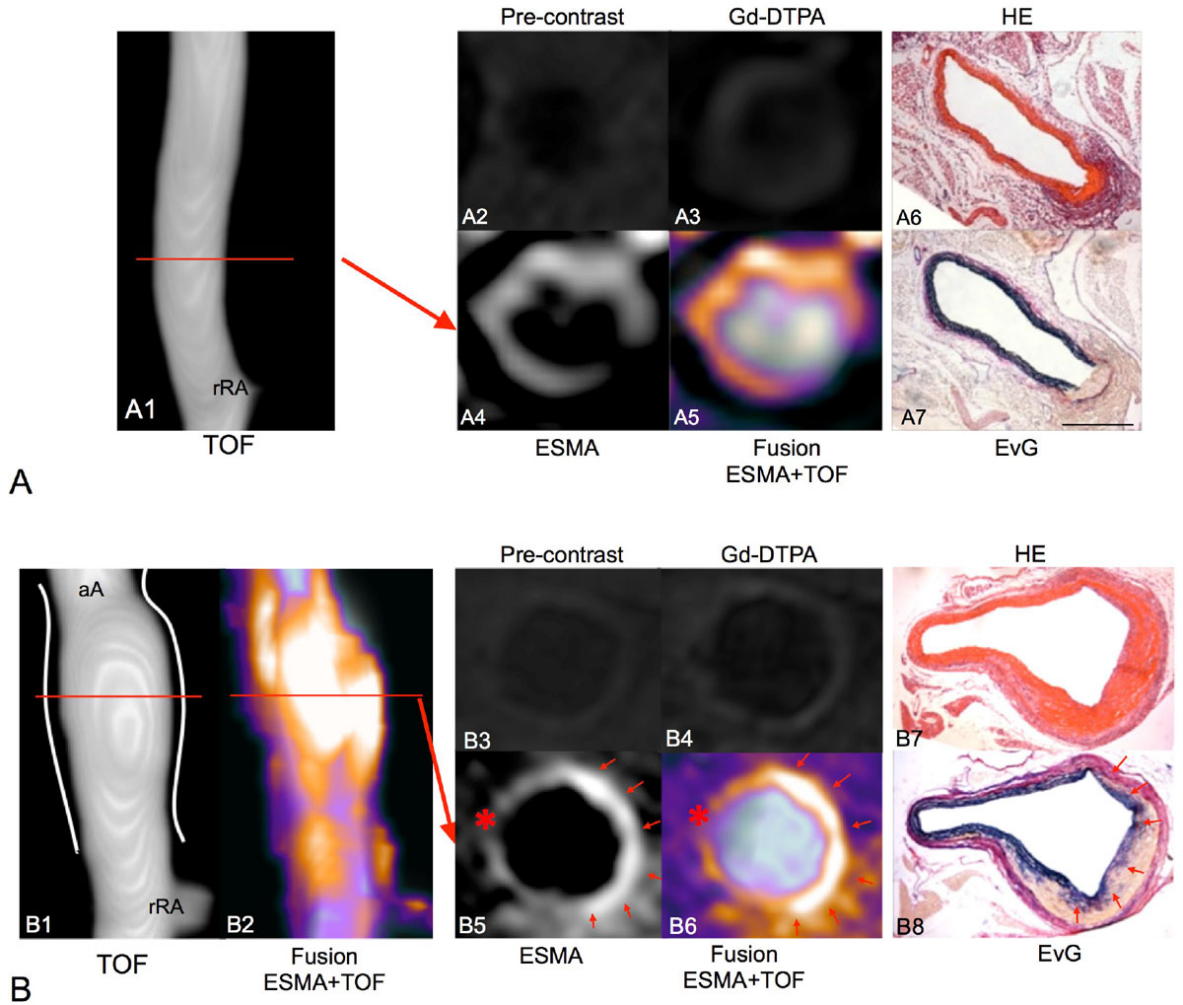
**A: Aortic angiogram of an ApoE-/- mouse 1 week after of Ang-II infusion without luminal dilatation or irregularities (A1)**. On pre-contrast (A2) and Gd-DTPA (A3) enhanced images only minor wall enhancement was observed. On ESMA-MRI the rupture of elastic laminae was clearly visualized in vivo (A4, A5), which was confirmed in corresponding histological sections (A6, A7). B: Aortic angiogram (B1) and fusion with ESMA scan (B2) 3 weeks after Ang-II. A significant increase in luminal diameter was observed resulting from the rupture of elastic laminae (B7, B8). We observed a strong increase in elastin formation at the site of the hematoma (B8, red arrows). Newly formed elastic fibers bridged the area in-between dissected elastic laminae (B8). This repair process was visualized by a strong enhancement on ESMA-MRI (B5, B6). On pre-contrast (B3) and Gd-DTPA (B4) images only minor enhancement was observed. The undisrupted wall showed only a moderate enhancement (*). aA: abdominal aorta, rRA: right renal artery.

**Figure 2:**
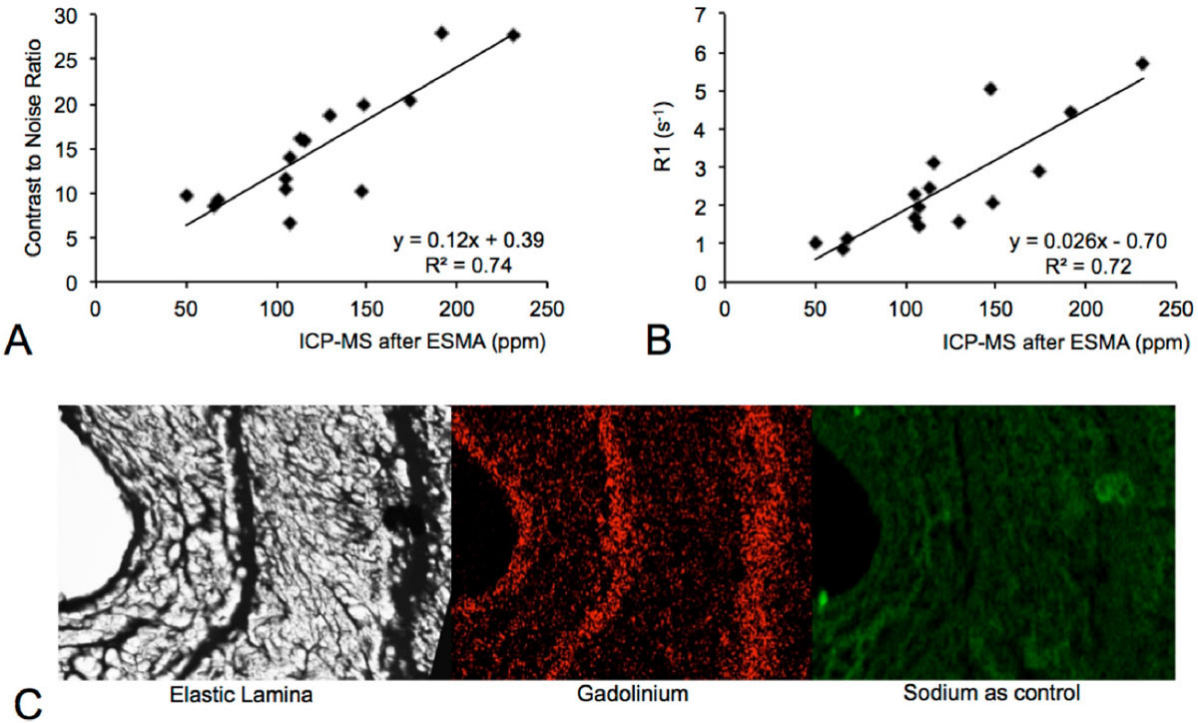
**A: The scatter plot shows a significant (p < 0.05) correlation between aneurysmal wall CNR and ICP-MS of collected aortas (n = 15)**. B: The scatter plot shows a significant (p < 0.05) correlation between aneurysmal wall R1 values and results from ICP-MS of collected aortas. After administration of Gd-DTPA, no significant increase in R1 could be measured compared to the pre-contrast scans. In line with this, ICP-MS demonstrated only a minor accumulation of Gd-DTPA in the aneurysmal wall after 4 weeks (12 ± 5 ppm). C: Typical gadolinium spectra (middle) measured in areas colocalizing with elastic fibers (left). No gadolinium spectra could be acquired in regions not associated with elastic fibers. The specific distribution of gadolinium was mapped in the arterial wall sample (middle). Colocalization of targeted gadolinium with elastic fibers could be found (n = 3). The mapping of sodium as control did not show a specific distribution pattern (right). Values are expressed as mean ± SD.

Imaging was performed with a spatial resolution of 100 μm on a clinical MRI system using clinical imaging protocols. After one week of Ang-II infusion, the elastin-specific probe enabled the clear in-vivo visualization of aortic rupture sites prior to the dilation of the lumen (Figure 1A). After 2 and 3 weeks of Ang-II infusion, a significant (p < 0.05) increase in luminal diameter was observed resulting from the rupture of elastic laminae (Figure 1B). We observed a strong increase in elastin formation at the site of the hematoma. Newly formed elastic fibers bridged the area in-between dissected elastic laminae. This repair process was clearly visualized and quantified by the elastin-specific probe. Using electron microscopy, co-localization of the probe with elastic fibers was found. The gadolinium concentration in AAAs increased as expression of elastin progressed. A significant correlation of CNR (p < 0.05) and R1 (p < 0.05) with ex-vivo gadolinium concentrations (ICP-MS) was found (Figure 2). After Gd-DTPA administration, no significant increase (p > 0.05) in R1 could be measured compared to pre-contrast scans.

## Conclusions

The elastin-specific molecular MR probe allows for the visualization and quantification of changes in elastin content at different stages of AAAs. This clinically translatable probe offers potential for the non-invasive detection of rupture sites prior to aortic dilation and subsequent monitoring of compensatory repair processes. This could enable a more accurate risk stratification and help guiding treatment decisions.

## Funding

This study was funded by the British Heart Foundation (PG/09/061).

